# Choroidal Thickness in Correlation with Axial Length and Myopia Degree

**DOI:** 10.3390/vision6010016

**Published:** 2022-03-02

**Authors:** Habibah Setyawati Muhiddin, Andi Ratna Mayasari, Batari Todja Umar, Junaedi Sirajuddin, Ilhamjaya Patellongi, Itzar Chaidir Islam, Andi Muhammad Ichsan

**Affiliations:** 1 Ophthalmology Department, Faculty of Medicine, Hasanuddin University, Makassar 90245, Indonesia; muhiddinhabibah@yahoo.co.id (H.S.M.); andiratna.maya@gmail.com (A.R.M.); bataritodjaumar@unhas.ac.id (B.T.U.); jedesirajuddin@yahoo.co.id (J.S.); itzarislam@unhas.ac.id (I.C.I.); 2 Physiology Department, Faculty of Medicine, Hasanuddin University, Makassar 90245, Indonesia; ilham_pt@yahoo.com

**Keywords:** choroidal thickness, myopia, axial length

## Abstract

Background: Myopia is a condition in which the visual images come to a focus in front of the retina of the eye. This disease is a major cause of visual disability, which presents in 108 million persons globally. Purpose: This study aims to determine the relationship between the degree of myopia, the axial length, and the choroidal thickness (CT). Methods: This is an observational analytical study that made use of a cross-sectional design. A total of 59 participants with refractive errors underwent treatment at Hasanuddin University Hospital and 116 eyes were measured and analyzed. The choroidal thickness was measured using the Enhance Depth Imaging OCT (EDI-OCT) tool, which is divided into nine observational areas. Furthermore, all data obtained were compared using statistical analysis, such as the one-way ANOVA and Pearson correlation test (*p* < 0.05). Results: There was a significant relationship between the choroidal thickness with axial length (*p* < 0.05) and myopia degrees (*p* < 0.05). Conclusions: The thickness of the choroid decreases with an increase in the axial length and degree of myopia, which further indicates that the higher the myopia degree, the thinner the choroidal vasculature.

## 1. Introduction

Myopia, also known as shortsightedness, is a major cause of visual disability around the world. In 1972 and 2004, the prevalence of myopia increased from 25% to 44% in the United States, while in Asia, the prevalence is approximately >80% [1]. In 2010, it was noted that the uncorrected refractive error was the major cause of vision impairment and the second most frequent cause of blindness, affecting 108 million persons globally. Furthermore, the cases of myopia are expected to increase by more than 5000 million by 2050 [2].

Myopia is more likely to occur in several conditions, such as young age (mostly 8 to 15 years old), hereditary person with myopic parent, and persons who work extensively with the eyes, such as microscopists, computer users, or university students [1,3]. According to the World Health Organization, the major cause of vision impairment and blindness around the world are macular degeneration, vitamin A deficiency, infectious disease, uncorrected refractive error with cataracts, and myopia [4].

Furthermore, myopia has been classified according to anatomical and pathological features, the age of onset, the rate of progression, the degree and theory of development. Physiological myopia occurs when the refractive components of the eye fail to correlate, unlike pathologic myopia (alternatively, malignant or degenerative myopia), which occurs when the optical system of the eye lies outside the limit of normal biological variations [5].

According to Okafor et al. (2009), the degree of myopia could be divided into three categories: Very low (<>−1.0 D), low (≤−1 to ≥−3.00 D), moderate (<−3.00 to ≥−6.00 D), high (<−6.00 D to ≥−10 D), and very high (<−10 D) myopias [6].

Myopia and refractive-error disorder may develop when there are irregular contributions of the ocular components to the eye structures. Four structures that contribute to the refractive status of a given human eye are aqueous and vitreous humor, cornea, and lens. When the lens and cornea fail to neutralize the axial length (AL) shortening or elongation, hyperopia or myopia may occur. Therefore, some parameters, such as the anterior chamber depth (ACD), corneal curvature, vitreous chamber depth, axial length, and lens thickness, are widely analyzed when studying eye diseases. However, among these components, more attention is paid to the axial length, which is the major parameter for both hyper myopia and myopia [7]. Furthermore, studies have shown that the alteration of environmental factors and the identification of genetic correlations may play a significant role in axial elongation, myopia progression, and future ocular complications [8,9].

Myopia is known to be the cause of multiple eye fatalities around the globe and investigation conducted on a particular population in various hospitals has shown that when the axial length or refractive error is ~26.5 mm or −8.00 D, the parapapillary atrophy and optic disc becomes enlarged gradually. However, when these values are higher, the prevalence of glaucomatous optic neuropathy and myopic retinopathy is increased. Myopia is identified as the excessive and continuous expansion of the axial length, resulting in a change in the secondary fundus, which leads to visual impairment, as well as choroidal neovascularization, retinal detachment, zonal areas of chorioretinal atrophy, myopic macular schisis, and hole [10].

In high myopic eyes, recent changes start in the choroid, therefore, studies have shown that the choroid is a very valuable structure that is required in the pathophysiology of high myopia [11]. The choroidal vasculature helps nourish the outer retina (including the photoreceptors), however, when there is a loss of the vascular tissue and an extreme thinning of the choroid, it leads to visual impairment and damage to the photoreceptors. The thickness of the choroidal is an essential parameter used for studying the pathogenesis of high myopia [12]. Furthermore, measurement of CT in vivo is suitable for determining the onset of diseases and their progression, which causes thinning of the choroidal [13]. The presence of lacquer cracks and choroidal neovascularization (CNV) is seen mostly in eyes with thinner macular choroids [14]. Therefore, this study aims to determine the relationship between the degree of myopia, the axial length, and the choroidal thickness.

## 2. Materials and Methods

### 2.1. Study Design

This is an observational analytical study that made use of a cross-sectional design. A total of 59 participants were included. There are 49 patients with refractive errors, who underwent treatment at Hasanuddin University Hospital, and 10 normal subjects as a control group. A total of 116 eyes were measured and analyzed. In this study, our criteria for recruiting subjects were patients aged 20–50 years, had a refractive error ≥0.5 D, did not suffer from anterior segment abnormalities during examination, no history of eye infection or eye surgery. Each patient who meets the criteria was asked to fill out and sign an informed consent form after then examined according to applicable standards.

### 2.2. Ophthalmology Examination

The examination was carried out by measuring the patient’s visual acuity and correction using a Snellen chart projector, trial lens, and retinoscopy. The results obtained from retinoscopy were in the form of a spherical equivalent (SE). The anterior segment of the eye was then examined with a slit lamp biomicroscope, then the patient was made to undergo an indirect funduscopic examination with a 90 D ocular lens to view the posterior segment of the eye. Subsequently, the length of the patient’s eyeball axis was obtained using A-Scan Ultrasound, which measures from the top of the cornea to the posterior segment, and the results were represented in millimeters (mm). The choroidal thickness was measured using the Enhance Depth Imaging OCT (EDI SD-OCT) tool, which is divided into nine observation areas. The type of OCT used is Heidelberg Spectralis^®^ OCT (3-mode), Germany. It was carried out by two technicians (two graders), then the results were blindly confirmed by the research team (HSM and AMI). The test was performed semi-automatically by drawing a perpendicular line between the outermost part of the hyper-reflective line that represents the RPE with the hypo-reflective line that represents the choroid-scleral surface using a calipers software tool.

### 2.3. Data Analysis and Interpretation

All results obtained were recorded and compared using SPSS for Windows version 24.0 with One-way ANOVA and Pearson correlation test (sig. *p* < 0.05), which is represented in tables and figures. The study was conducted in accordance with the Declaration of Helsinki and approved by the Institutional Ethics Committee for Medical Research of Hasanuddin University (Approval No.: 751/UN 4.6.4.5.31/PP36/2021).

## 3. Results

The degree of myopia is divided into three categories: Low (≥−3.00 dpt), moderate (<−3.00 to ≥−6.00 dpt), and high (<−6.00 dpt) myopias. This was compared with the choroidal thickness found in various areas of the macula. The result obtained showed a significant difference except for the 1.5 mm temporal region (T3) (Table 1). This result indicates a significant relationship between the choroidal thickness and the degree of myopia. A high degree myopia showed a thinner choroidal vasculature.

**Figure 1:**
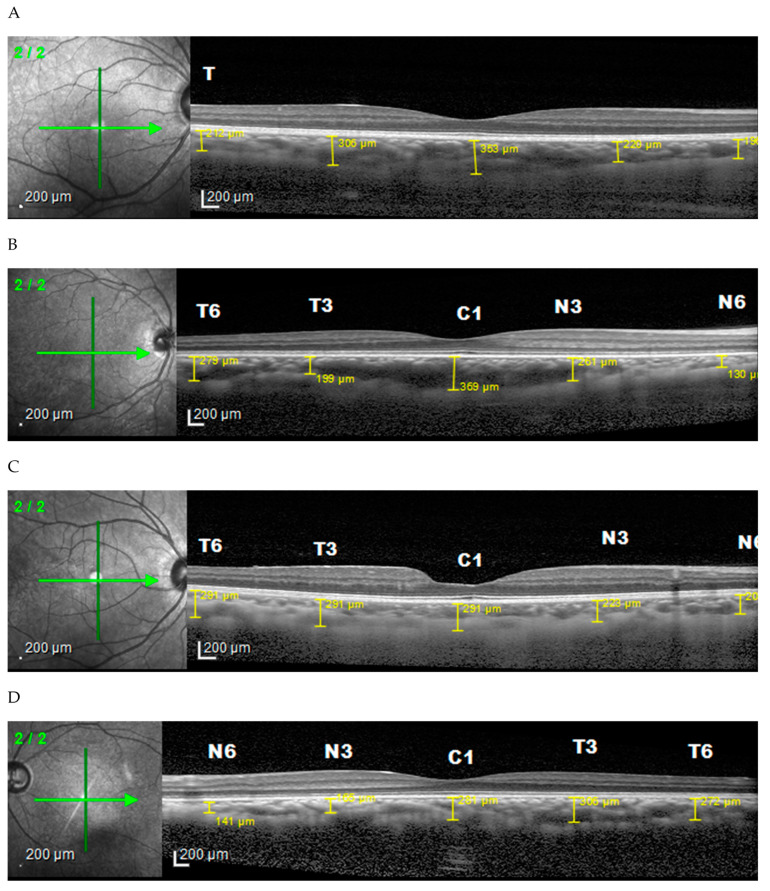
Choroidal thickness based on degrees of myopia horizontally. (**A**) Normal subject, (**B**) low myopia, (**C**) moderate myopia, (**D**) high myopia.

**Figure 2:**
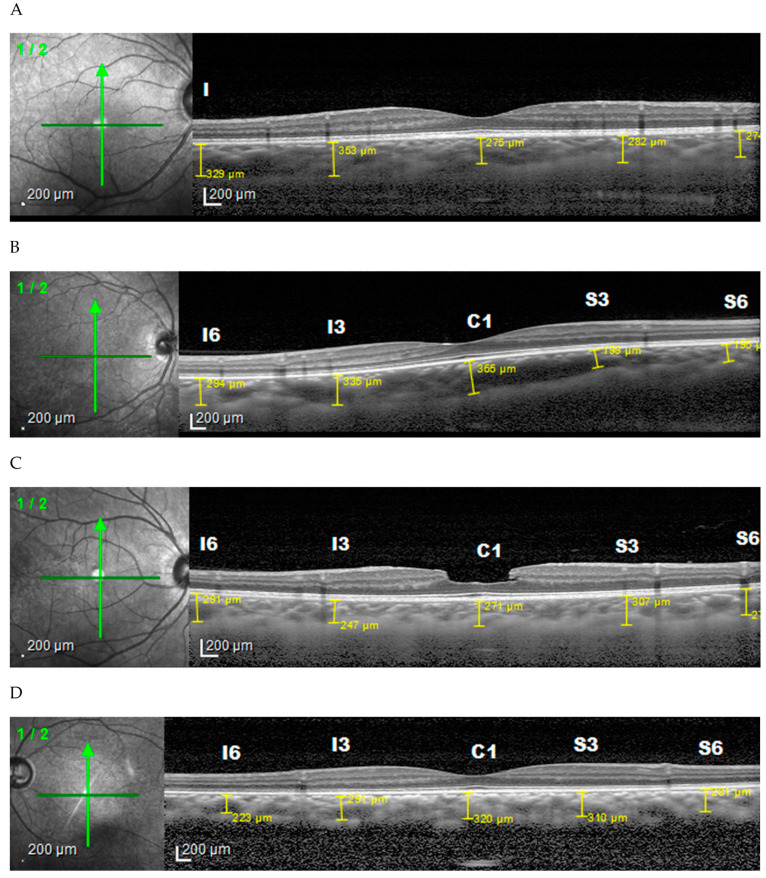
Choroidal thickness based on degrees of myopia vertically. (**A**) Normal subject, (**B**) low myopia, (**C**) moderate myopia, (**D**) high myopia.

From the horizontal image (Figure 1), the choroid found in the low myopia is thickest in the subfoveal unlike in medium and high myopia where it is thickest in the temporal. The thinnest area in all groups was the nasal area. Vertically (Figure 2), in low and moderate myopia, the choroid is thickest in the superior area, while in high myopia it is thickest in the subfoveal area. The thinnest area in each group was the inferior region. Comparison of the choroidal thickness based on the axial length showed a significant difference (*p* < 0.05), except for the superior region (S6).

**Figure 3:**
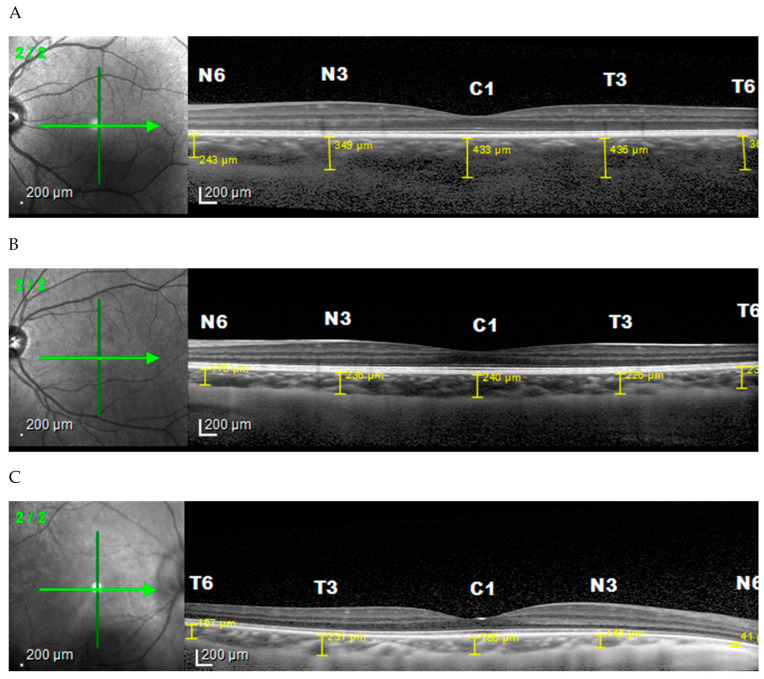
Choroidal thickness based on axial length horizontally. (**A**) Normal axis, (**B**) medium axis, (**C**) long axis.

**Figure 4:**
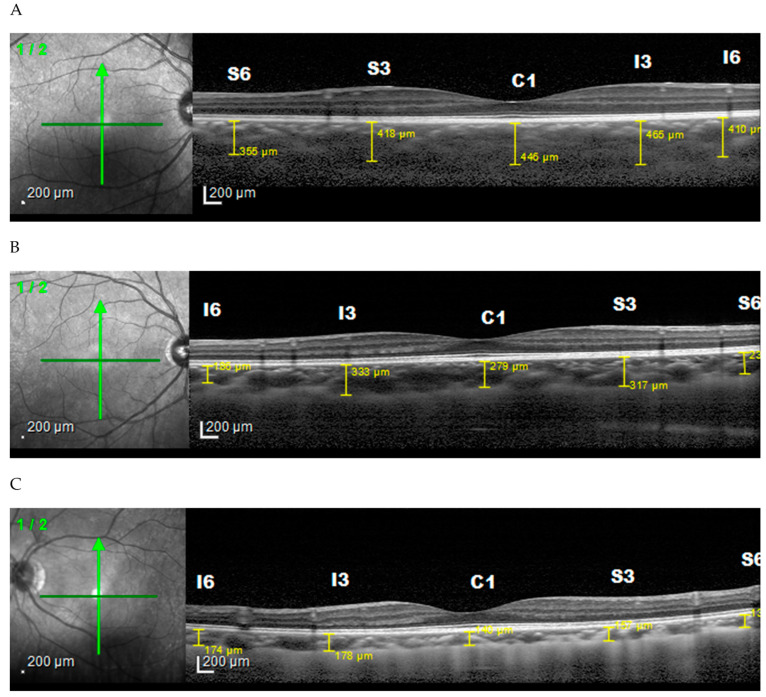
Choroidal thickness based on axial length vertically. (**A**) Normal axis, (**B**) medium axis, (**C**) long axis.

The mean choroid thickness of the normal (<22.5 mm), medium (22.5 to 25.0 mm), and long (>25.5 mm) axis in the horizontal section (Figure 3) were thickest in the fovea, while the thinnest in each group was found in the nasal area. Furthermore, vertical measurement (Figure 4) on the normal axis showed that the thickest area is on subfoveal area while on the medium and long axis it is thickest in the superior area. The thinnest area in the normal and medium axis groups was the inferior region, while for the long axis it was the subfoveal area.

Table 2 showed that there is a significant correlation between choroidal thickness and axial length (*p* < 0.05), except on S6 area. This indicates that the thickness of the choroid decreases with increasing axial length.

Table 3 shows the correlation coefficient between the axial length of the eyeball and the thickness of the choroid (r = 0.270–453), which is higher than the degree of myopia (r = 0.230–407).

**Figure 5:**
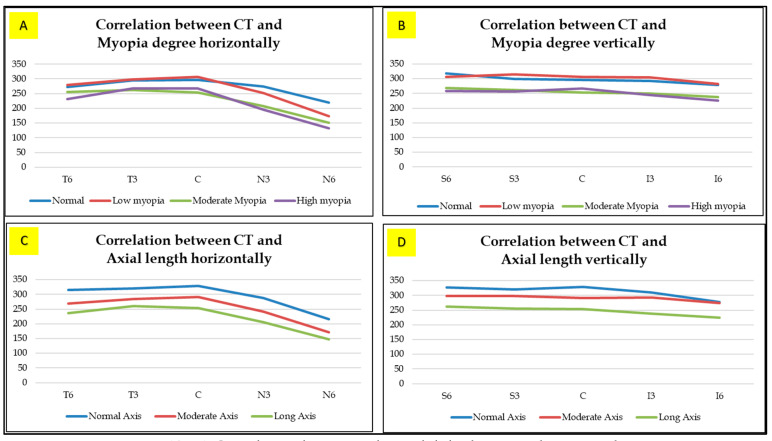
(**A**,**B**) Correlation between choroidal thickness and myopia degree. (**C**,**D**) Correlation between choroidal thickness and axial length.

The choroidal thickness showed a significant relationship (*p* < 0.05) with the axial length and degree of myopia found in various areas of the macula and this has a negative correlation coefficient. Therefore, the longer the axis of the eyeball, the higher the degree of myopia, which will lead to choroidal thinning (Figure 5).

## 4. Discussion

Historically, some opticians thought that myopia was a hereditary abnormality, whereas others imagined it to be environmentally induced. However, several studies conducted on animals and humans over the last four decades suggest that the occurrence of myopia is controlled by both genetic and environmental factors [3].

The comparison between the degrees of myopia and choroidal thickness showed a significant correlation (*p* < 0.05) that indicates that a higher degree of myopia will lead to thinning of the choroidal layer. Moreover, the mean choroidal thickness obtained by OCT examination was based on the degree of myopia found in the horizontal area. The low degree myopia was found to be thickest in the subfoveal area, while the moderate and high degree myopia was thickest in the temporal region. The thinnest choroid was found in the nasal area of all degrees of myopia. A study by Shin (2012) reported that the choroid became thinner about 13.62 μm for each diopter of refractive error and also decreased about 1.31 μm for each year of age [15].

Based on studies conducted, it is known that the thinning of the choroid reduces ischemia and perfusion of the choroid, which leads to the upregulation of the angiogenic factors in the eyes and this may also lead to the formation of myopic choroidal neovascularization and other features of macular degeneration [16].

In a cross-sectional study by El-Shazly et al. (2017), Macular CT was measured in different degrees of myopia and in normal control eyes, and a similar result was obtained, which is significantly lower in myopes than in emmetropes. Moreover, it varies by location, where the thickest CT in low myopic eyes is found in the subfoveal area, while the thinnest is located in the nasal region. However, for eyes with moderate myopia, the thickest is found in the temporal region, while the thinnest region remained in the nasal direction [17].

Another study by Deng et al. (2018) showed that the mean CT in the perifoveal, parafoveal, and central foveal regions where 215 ± 50 µm, 227 ± 60 µm, and 229 ± 65 µm, respectively, while the mean spherical equivalent (SE) of the patient was −1.71 ± 2.22 diopter (D) (range from −7.63 to 4.25 D). Furthermore, the mean global peripapillary choroidal thickness (PPCT) was 136 ± 33 µm [18].

Based on the result obtained from different studies carried out on animals, an alteration in the thickness of the choroid may occur when maintaining a clear vision. Earlier studies on macaques, marmosets, and chicks have led to the hypothesis which states that the thickening of the choroid may occur when myopic defocus is induced due to changes in the position of the retina when maintaining a clear vision. This is possible because, in myopic defocus, the retina is at the back of the image plane, so when thickening of the choroid occurs, it moves the retina forward [19].

Furthermore, this study also indicates that an increase in axial length leads to a decrease in the thickness of the choroid (Table 2). Moreover, the choroidal thickness showed a significant relationship (*p* < 0.05) between the axial length and the degree of myopia found in various areas of the macula that has a negative correlation coefficient. Therefore, this indicates that the longer the axis of the eyeball and the higher the degree of myopia, the more choroidal thinning will occur.

In general, the Axial length increases rapidly at the early stage of life but slowly in adulthood and decreases in old age [7]. According to Lee et al. (2020), the best-corrected visual acuity (BCVA), baseline axial length, anterior chamber depth, and age were significantly associated with changes in axial length (*p* = 0.005, *p* < 0.001, *p* = 0.006, and *p* = 0.045, respectively) [19].

Furthermore, the choroid may stimulate axial growth by regulating the remodeling of the scleral extracellular matrix, which is important for emmetropization during eye formation. In experimental animals induced with hyperopia and myopia, a change in the thickness of the choroid exceeds that of axial length and scleral remodeling [12].

According to histologic, clinical, and population-based investigations, an increase in axial elongation led to a significant thinning of the choroid. Furthermore, in emmetropic subjects, the mean CT was 250 μm, while in highly axially myopic patients, it decreased to <30 μm. Therefore, this indicates that an increase in the axial elongation led to a decrease in the distance between the Bruch membrane and sclera [17].

Based on Jin et al. (2016), the myopic retinas were thinner than those of emmetropic or hyperopic subjects, especially in the superior parafoveal and all four perifoveal subfields (*p* < 0.05). However, the results of previous studies on factors influencing the thickness of the ganglion cell layer and nerve fiber layer have been conflicting. While some suggested that the thickness of the ganglion cell layer and peripapillary nerve fiber layer is correlated with spherical equivalent refraction and axial length in adults, others did not observe this relationship [12].

Karahan (2013) reported that choroidal change plays a major role in the development and progression of many retinal diseases. Thickening of the choroid could affect the nutrition supply to the retina, because the outer retina layer is nourished by the choroidal vasculature. Thus, choroidal thickness provides useful information to clinicians [20].

An increase in the axial length led to an increase in the retina thickness, which aided in blood supply. This, however, caused an increase in length of the ocular axis, resulting in a compensatory thickening of the choroidal capillary layer in the fovea centralis and an increase in the number of capillaries and volume. Moreover, an increase in the myopia degree will cause the axial length of the eye to increase as well. However, the retina may fail to compensate, causing the capillary layer and choroid to become thinner [21].

## 5. Conclusions

There was a significant correlation between the degree of myopia and choroidal thickness. Therefore, a higher degree of myopia will cause a decrease in the thickness of the choroid. Furthermore, it is necessary to screen for choroidal thickness in myopic patients using SD-OCT, especially in moderate and high degrees, to prevent complications because a significant increase in myopia degree will lead to a decrease in the choroidal thickness. Moreover, it is necessary to measure the length of the eyeball axis in myopic patients because a decrease in choroidal thickness is significant to the elongation of the eyeball. All of these examinations are to prevent some further complications, such as the progressivity of myopic choroidal neovascularization (CNV), which will eventually lead to worsening of vision.

## Figures and Tables

**Table 1:** Comparison of choroidal thickness and the degrees of myopia.

Choroidal Thickness Based on Area (Mean ± SD) µm		Myopia Degree (D)			*p* *
	Normal (*n* = 20)	Low Myopia (*n* = 50)	Moderate Myopia (*n* = 31)	High Myopia (*n* = 15)	
C = Sub Fovea	296.70 ± 69.62	307.52 ± 79.30	253.39 ± 64.84	267.33 ± 104.01	0.021
T6 = Temporal 3	273.35 ± 46.82	280.22 ± 76.18	255.06 ± 47.20	231.07 ± 59.52	0.047
T3 = Temporal 1.5	295.45 ± 66.94	298.12 ± 78.30	262.58 ± 54.02	267.80 ± 83.84	0.136
N3 = Nasal 1.5	274.15 ± 71.70	252.50 ± 64.89	208.23 ± 48.70	196.07 ± 89.16	0.001
N6 = Nasal 3	219.25 ± 77.72	173.00 ± 50.72	151.06 ± 47.20	132.33 ± 77.61	0.001
S6 = Superior 3	319.90 ± 84.54	306.02 ± 72.50	269.13 ± 56.69	258.67 ± 89.28	0.039
S3 = Superior 1.5	299.40 ± 76.45	314.18 ± 80.06	261.52 ± 52.95	257.73 ± 93.38	0.008
I3 = Inferior 1.5	293.95 ± 77.33	307.12 ± 71.74	249.97 ± 62.72	244.73 ± 76.31	0.001
I6 = Inferior 3	278.50 ± 86.19	282.04 ± 62.99	237.84 ± 47.31	226.00 ± 65.93	0.003

* One-way Anova test.

**Table 2:** Comparison of choroidal thickness and axial length.

Coroidal Thickness Based on Area (Mean ± SD) µm	Axial Length (mm)			*p* *
	Normal (*n* = 6)	Moderate (*n* = 81)	Long (*n* = 29)	
C = Sub Fovea	329.50 ± 27.28	291.62 ± 76.94	253.28 ± 86.84	0.029
T6 = Temporal 3	315.17 ± 75.89	269.99 ± 62.44	236.34 ± 55.72	0.006
T3 = Temporal 1.5	320.00 ± 37.06	285.42 ± 71.87	261.76 ± 70.54	0.120
N3 = Nasal 1.5	288.17 ± 42.97	241.38 ± 67.44	206.52 ± 71.63	0.010
N6 = Nasal 3	216.83 ± 60.60	171.73 ± 60.47	147.93 ± 62.65	0.029
S6 = Superior 3	327.00 ± 40.71	298.06 ± 78.31	262.45 ± 72.23	0.049
S3 = Superior 1.5	320.83 ± 36.40	298.53 ± 76.42	256.14 ± 79.89	0.024
I3 = Inferior 1.5	310.50 ± 45.47	293.37 ± 73.36	239.79 ± 68.67	0.002
I6 = Inferior 3	278.33 ± 64.76	274.07 ± 67.91	225.48 ± 56.06	0.003

* One-way ANOVA test.

**Table 3:** Correlation between axial length, degree of myopia, and choroidal thickness in various regions.

Choroidal Thickness Based on Area (Mean ± SD) µm	Axial Length (mm)		Myopia Degree (D)	
	Coefficient Correlation (r)	*p* *	Coefficient Correlation (r)	*p* *
C = Sub Fovea	−246	0.008	−175	0.060
T6 = Temporal 3	−293	0.001	−180	0.053
T3 = Temporal 1.5	−190	0.041	−124	0.186
N3 = Nasal 1.5	−278	0.003	−347	<0.001
N6 = Nasal 3	−238	0.010	−368	<0.001
S6 = Superior 3	−288	0.014	−248	0.007
S3 = Superior 1.5	−249	0.007	−227	0.014
I3 = Inferior 1.5	−310	0.001	−284	0.002
I6 = Inferior 3	−289	0.002	−299	0.001

* Pearson correlation test.

## Data Availability

The data that support the findings of this study are available from the corresponding author upon reasonable request.
